# Transcriptional analysis of islets of Langerhans from organ donors of different ages

**DOI:** 10.1371/journal.pone.0247888

**Published:** 2021-03-12

**Authors:** Peter Seiron, Anton Stenwall, Anders Hedin, Louise Granlund, Jonathan Lou S. Esguerra, Petr Volkov, Erik Renström, Olle Korsgren, Marcus Lundberg, Oskar Skog

**Affiliations:** 1 Department of Immunology, Genetics and Pathology, Uppsala University, Uppsala, Sweden; 2 Department of Clinical Sciences-Malmö, Lund University Diabetes Centre, Malmö, Sweden; 3 Institute of Medicine, University of Gothenburg, Gothenburg, Sweden; La Jolla Institute for Allergy and Immunology, UNITED STATES

## Abstract

Insulin secretion is impaired with increasing age. In this study, we aimed to determine whether aging induces specific transcriptional changes in human islets. Laser capture microdissection was used to extract pancreatic islet tissue from 37 deceased organ donors aged 1–81 years. The transcriptomes of the extracted islets were analysed using Ion AmpliSeq sequencing. 346 genes that co-vary significantly with age were found. There was an increased transcription of genes linked to senescence, and several aspects of the cell cycle machinery were downregulated with increasing age. We detected numerous genes not linked to aging in previous studies likely because earlier studies analysed islet cells isolated by enzymatic digestion which might affect the islet transcriptome. Among the novel genes demonstrated to correlate with age, we found an upregulation of *SPP1* encoding osteopontin. In beta cells, osteopontin has been seen to be protective against both cytotoxicity and hyperglycaemia. In summary, we present a transcriptional profile of aging in human islets and identify genes that could affect disease course in diabetes.

## Introduction

Efforts are currently being directed to determine the effects of aging on human beta-cell biology and physiology. Glucose tolerance is decreased in the aging human, as a result of an increase in resistance to insulin signaling in the peripheral tissue and impaired insulin secretion [1–3], implying that beta cells become less functional with age. The progressive decline in the secretory capabilities of the beta cell has been explained in several different ways, with two of the most important points thus far being a compromised cell physiology and a reduced number of physiologically active beta cells [4].

Indeed, in a recent paper, Westacott et al. reported an age-related decline in the coordination of Ca^2+^ signaling between beta cells within isolated islets that is associated with impaired insulin dynamics [5]. Furthermore, the physiological regulation of the membrane-bound K^+^ transporter crucial to insulin secretion has been found to undergo an age-dependent decline in rodents suggesting an age-dependent dysfunction in beta-cell ion homeostasis [6]. Beta cells from both rodents and humans have been suggested to show an age-related decline in mitochondrial function, resulting in disrupted Ca^2+^ dynamics and impaired insulin secretion [7].

The concept of a gradual loss of physiologically active beta cells in the aging human has been subject to debate. Several important findings point toward an age-related decline in replicative ability, as reviewed by Kushner [8]. Key studies investigating postnatal beta cell turnover have shown very little evidence of replicating beta cells in adults when Ki67 is used as a marker for cell division [9, 10] or when age-related accumulation of lipofuscin bodies in the beta cells is used as an indicator of replication [11]. A study measuring the incorporation of iodo-deoxyuridine (IdU) into beta cells from patients receiving thymidine analogs during clinical trials for cancer therapy has found no signs of replication in subjects older than 30 years [12]. Loss of beta-cell mass as a consequence of an age-dependent increase in beta-cell apoptosis has been suggested as another key component of the decline in secretory function, as has the loss of beta-cell identity when the beta cells lose their secretory phenotype [13].

Aging has previously been shown to affect gene expression patterns on a transcriptional level in mouse islets. One of the genes that has been shown to be up-regulated is *CDKN2A*, which encodes the negative cell cycle regulator p16^INK4A^ [14, 15]. Some of these findings were later confirmed by Helman et al., who found p16^INK4A^ to be up-regulated in isolated islets from adult mice when compared to islets from young animals [16].

Recent studies that have investigated the effects of aging have been conducted on isolated rodent islets [17] or isolated human islets digested into single cells [18]. The aim of this study was to characterize the transcriptome in human islets retrieved from high-quality pancreatic tissue samples from deceased organ donors of 1 to 81 years of age using laser capture microdissection (LCM) that minimizes changes in islet physiology in order to determine whether aging induces specific gene expression patterns in human.

## Materials and methods

### Ethics

This study was made possible through the donation of human tissue from deceased organ donors. The consent to organ donation for transplantation and research was obtained verbally from the next of kin by the physician in charge or obtained from an online database (https://www.socialstyrelsen.se/donationsregistret/) in those instances in which this information was available. The process was documented in full accordance with Swedish law and applicable regional standard practices. All tissue included in the study was procured, documented, stored, and analyzed in accordance with approval from the regional Ethics Committee in Uppsala (Dnr 2009/371/2 and Dnr 2015/444).

### The study population

This research was based on the study of frozen pancreatic tissue samples from deceased organ donors procured within the Nordic Network for Islet Transplantation. Initially, six donors aged 12 years old and younger (children) were included in the study. In addition, the same number of donors (n = 6) was initially included for each of the following age groups: 13–18 years (adolescents), 25–35 years (young adults), 50–60 years (middle-aged), and > 70 years of age (elderly). The most recent organ donors with a normal HbA1c (<6.0%, 42 mmol/mol) and without obesity (defined as BMI <30 kg/m^2^) were included in each age group. Also, raw data from seven non-diabetic donors used as controls in a previous study (Granlund, L. *et al*. Manuscript in preparation) and acquired in an identical fashion were included in the study. Four donors were subsequently excluded because of technical issues (see section *Transcriptome analysis* below). Group characteristics of the 33 donors in the transcriptome analyses are summarized in Table 1.

**Table 1:** Group characteristics of the donors where LCM and transcriptome analysis were successful.

	Children	Adolescents	Young Adults	Middle-aged	Elderly
Mean age, years, (range)	5.8 (1–12)	15.9 (13–18)	29.2 (21–35)	56.3 (50–63)	75.9 (72–81)
Mean HbA1c*, %, (range)	5.3 (5.2–5.4)	5.6 (5.4–5.8)	5.3 (4.8–5.7)	5.8 (5.4–6.3)	5.7 (5.5–5.8)
Mean HbA1c, mmol/mol, (range)	34.5 (33–36)	37.7 (36–40)	34.4 (29–39)	40 (36–45)	39.2 (37–40)
Number of donors, *n*	5	8	6	8	6
Male, *n*	5	4	5	4	3
Female, *n*	0	4	1	4	3
Mean BMI, kg/m2, (range)	16.42 (13.9–20.4)	22.4 (16–28.9)	25.1 (21.8–28)	23.9 (19.4–29)	24.2 (22.3–26.1)
Mean SI, high/low, (range)	n/a	10.1	21.4 (3.9–64.3)	8.7 (2.3–14.4)	4.6 (2.0–6.3)

*HbA1c was available from 4/5 children, 3/8 adolescents, 5/6 young adults, 8/8 middle-aged 6/6 elderly; SI = stimulation index.

### Glucose-stimulated insulin secretion

In those cases in which the pancreas of the donor had also been used for clinical islet isolation (1 adolescent donor, 4 donors in the age group 25–35 years, all 6 donors aged 50–60 years, and 4 donors aged >70 years), data were available from a routine assessment of glucose-stimulated insulin secretion from 20 handpicked islets using a dynamic perfusion system, Suprafusion 1000 (BRANDEL, Gaithersburg, MD). In brief, the islets were sequentially perfused with low (1.67 mM) glucose for 48 min, high (20 mM) glucose for 42 min, and then low glucose again. Fractions were collected at 6-min intervals, and the secreted insulin was measured by ELISA (Mercodia, Uppsala, Sweden). The dynamic stimulation index at each time point was calculated by normalizing the insulin concentration in each fraction to the mean basal insulin secretion at low glucose from the same islet sample.

### Tissue preparation

All pancreata were transported in cold preservation solution to Uppsala University Hospital for the purpose of islet isolation. Before islet isolation according to a protocol described previously [33], pancreatic tissue samples were taken at the site adjacent to the clamp fixating the catheter used to inject collagenase in the pancreatic duct. The tissue samples were snap frozen in liquid nitrogen, and stored at -80°C. The pancreatic tissue samples were taken, one at a time, from storage at –80°C and processed through a Leica CM1860 UV cryostat (Leica, Wetzlar, Germany); the tissue was sectioned at 10 μm at -22°C and placed on either an Arcturus PEN Membrane slide that had been UV treated for 12 min (Thermo Fischer Scientific, MA, USA) for downstream LCM or on a Superfrost Ultra Plus object glass (Thermo Fischer Scientific, MA, USA) for subsequent immunohistochemical (IHC) staining. The PEN membranes were immediately stored at -80°C until used for LCM. In total, 13 consecutive sections were taken from each tissue sample, with 10 sections being placed on PEN membranes and 3 sections on object glasses. IHC was performed by utilizing a standardized insulin staining protocol. Reagents were bought from Dako (Agilent, CA, USA) if not otherwise specified. In brief, sections were fixed in 4% paraformaldehyde, blocked for intrinsic peroxidase activity with peroxidase blocking reagent, and then blocked using 5% goat serum diluted in TRIS-buffered saline supplemented with 0.05% Tween-20. Sections were stained for insulin (1:200, polyclonal guinea pig, clone A0564) for 1 hr and visualized using the DAKO EnVision+ System-HRP (DAB) with anti-rabbit secondary antibody (clone K4003).

### Laser capture microdissection and tissue processing

The PEN membranes were quickly removed from -80°C, one at a time, and thawed and dehydrated utilizing a standard EtOH protocol with subsequent thawing steps in 75% EtOH for 30 sec at -20°C, followed by 95% EtOH for 60 s and 100% EtOH for 60 s at room temperature. This EtOH protocol was immediately followed by a 4-min dehydration step in xylene at room temperature. LCM was carried out on an Arcturus XT LCM instrument. The islets of Langerhans were located through autofluorescence, and their location was confirmed by inspecting the corresponding object glass stained for insulin. The islets were captured on Arcturus CapSure HS LCM Caps (Applied BioSystems, CA, USA), and the caps were incubated immediately after LCM in 20 μl of RLT Buffer Plus (Qiagen, Hilden, Germany) containing 1% beta-mercaptoethanol. All samples were stored at -80°C prior to down-stream processing. RNA was extracted from at least 21 pooled islet sections per donor with AllPrep DNA/RNA micro kit (Qiagen). This generated RNA for downstream transcriptome analysis.

### Transcriptome analysis

Total RNA was reverse-transcribed using the Ion AmpliSeq Transcriptome Human Gene Expression kit according to the manufacturer’s instructions (Life Technologies). Expression data for 20,813 human genes were generated by transcriptome sequencing using an Ion AmpliSeq Human Whole Transcriptome kit according to standard procedures described previously [19]. The sequencing depth was 7–13 M mapped reads per sample and the mapping rate was >94%. One donor failed post-sequencing QC testing and was removed from further analysis.

Analysis of differential gene expression was performed in R version 3.5.3 using edgeR [20, 21].

The gene counts were preprocessed using the filterByExpr function in edgeR with default settings and using five samples as the minimum to pass filtration.

Normalization factors for each library were found using the calcNormFactors function in edgeR to calculate TMM-normalization factors [22]. TMM-adjusted counts per million (CPM)-normalized counts were extracted using the cpm-function and used for data exploration in Omics Explorer version 3.6 (Qlucore, Lund, Sweden). In an initial principal component analysis, three donors were excluded as outliers based on visual inspection. A new normalization was performed on the remaining 33 donors Raw counts for the 30 donors initially collected for this study as well as normalized counts for all 33 donors in the final analysis are available at GEO series accession number: GSE165121. Raw counts added from the previous study are available at GSE162689. Normalized counts are available in S1 Table.

Then dispersion was estimated with the estimateDisp function. A generalized linear model was then fitted using glmQLFit. As the libraries came from two different batches, that on an MDS plot clustered separately, the design corrected for this known batch-effect. The genes were analysed using age in years as a continuous numerical variable. Differential gene expression was assessed with the glmQLFtest function in edgeR, utilizing a quasi-likelihood test. Fold change is calculated per year. FDR was determined using the Benjamini Hochberg procedure. A previously published RNAseq data set [23] of gene expression in isolated islets and isolated exocrine tissue was used to exclude an additional 96 genes with >10-fold higher expression in exocrine tissue, as small contaminations would risk skewing the data.

Heat maps, principal component analysis and scatter plots were made using Omics Explorer version 3.6.

### Pathway enrichment analysis

Gene set enrichment analysis (GSEA) was performed using GSEA software 4.0.3 (Broad Institute, USA) with a preranked list of -log10(p-value) multiplied by the sign of log fold change [24]. Gene sets containing between 15 and 500 genes from GO [25] and REACTOME [26] were used. The analysis was run with 1000 permutations and a weighted enrichment statistic.

Clustering, annotation, and visualization of enriched gene sets were performed using EnrichmentMap suite [27] within Cytoscape 3.7.2 software [28]. Nodes were sized according to normalized expression score (NES), with size increasing from NES 1.5 and 2.5. Annotations were manually curated.

Functional enrichment analysis was performed using g:GOst within g:Profiler (https://biit.cs.ut.ee/gprofiler/gost) [29] using only differentially expressed genes of FDR<20% and log2 fold change >1.5 over 80 years. Significance using g:GOst was determined using their proprietary g:SCS correction and thresholded at p<0.05.

## Results

### Dynamic glucose-stimulated insulin secretion from islets from donors of different ages

**Fig 1:**
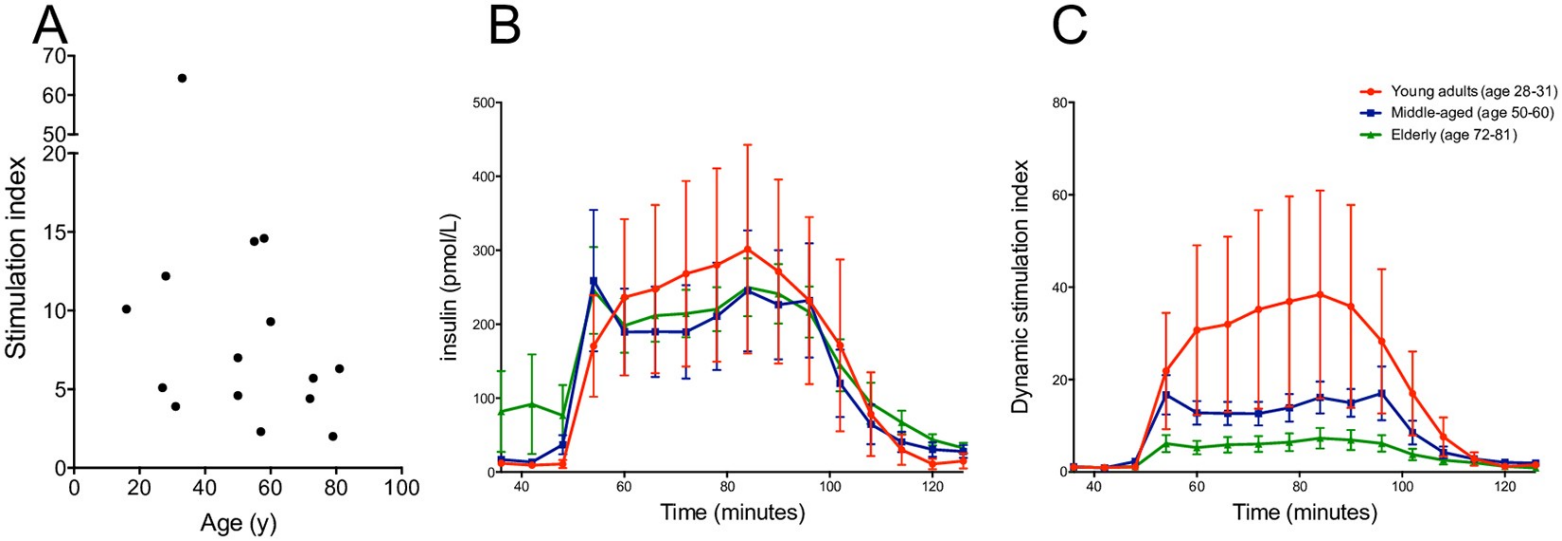
Glucose-stimulated insulin secretion in isolated islets assessed in a dynamic perifusion system. Twenty handpicked islets were sequentially perifused with low (1.67 mM) glucose during minutes 1–48, high (20 mM) glucose during minutes 48–90, and then low glucose again during minutes 90–120. Fractions were collected at 6-min intervals, and the secreted insulin was measured by ELISA. The stimulation index (ratio mean secretion at high glucose vs. mean secretion at low glucose) for each donor is shown in (**A**). In (**B**) the concentration of insulin in the collected fractions is shown, and the dynamic stimulation index at each time point is displayed in (**C**). The dynamic stimulation index at each time point was calculated by normalizing the insulin concentration in each fraction to the mean basal insulin secretion at low glucose from the same islet sample. Data available from 1/8 adolescents, 4/6 young adults, 6/8 middle-aged. 4/6 elderly.

Islets from all donors from which islets had been isolated responded to high glucose with increased insulin secretion, with the stimulation index ranging from 2.0 to 64.3 (Table 1 and Fig 1A). In absolute values, the insulin secretion was similar in all groups, with a slightly increased basal secretion in the oldest donors (Fig 1B) resulting in a lower dynamic stimulation index (Fig 1C).

### Global gene expression

**Fig 2:**
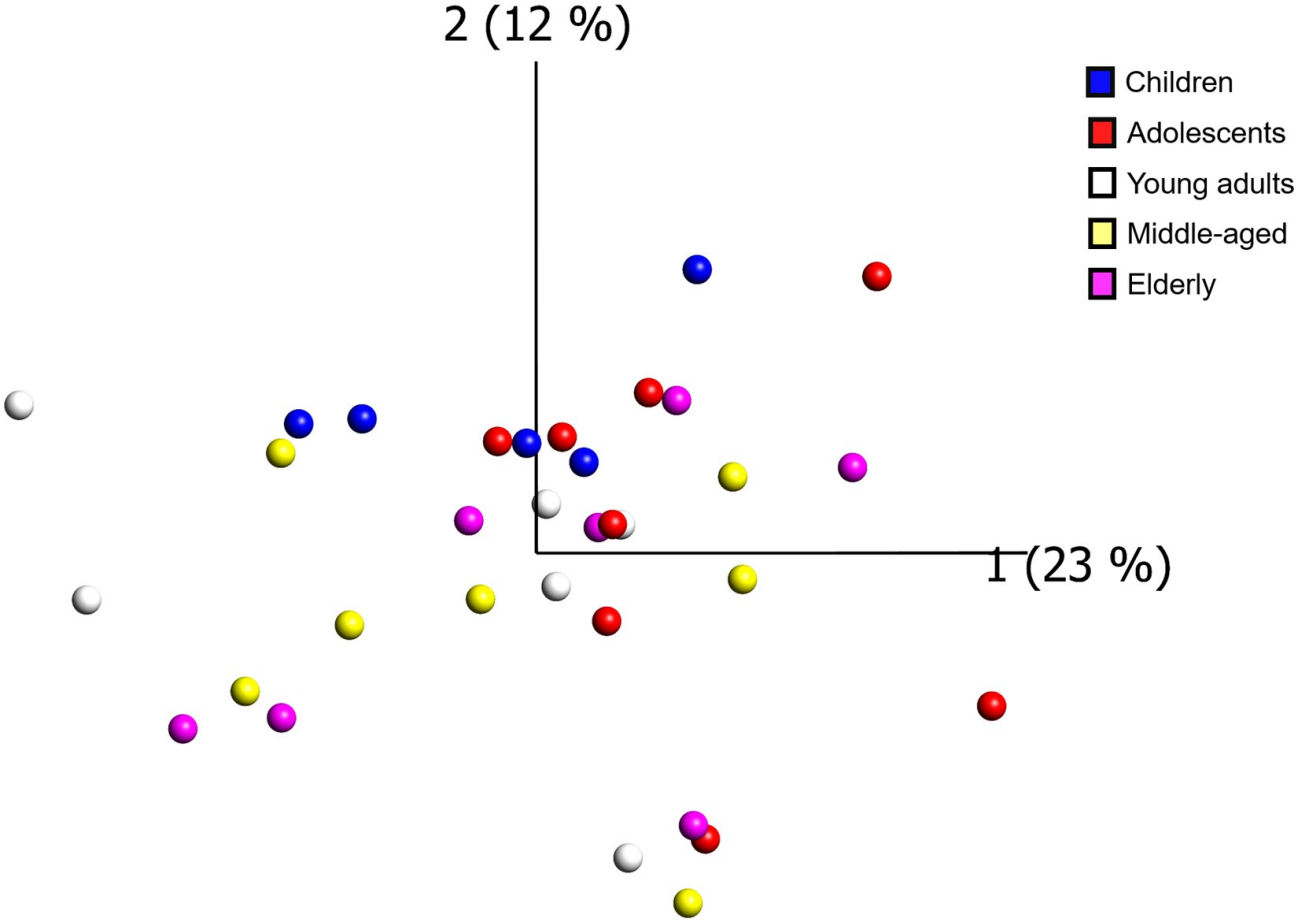
Global gene expression across all samples. Principal component analysis of all expressed genes did not show any evident clustering based on donor age. Each sample is represented as a dot with a color corresponding to the age group of the donor (blue = children (1–12 years), red = adolescents (13–18 years), white = young adults (21–35 years), yellow = middle-aged (50–63 years), magenta = elderly (72–81 years)).

14,794 genes passed filtering, by having a minimum of 10 CPM-corrected counts in at least 5 samples. The expression of these genes did not show any evident clustering based on donor age using principal component analysis (Fig 2). Expression data for all 14,794 analyzed genes are provided in S1 Table.

### Differential gene expression and age

Differential expression was determined with a generalized linear model fitted with age as a continuous variable. Three hundred forty-six (346) genes were found that co-varied significantly with age (FDR <20% and with log2 fold change >1.5 across 80 years of age) of these 270 genes were upregulated and 76 downregulated with increasing age (S2 Table).

### Pathway enrichment analysis

**Fig 3:**
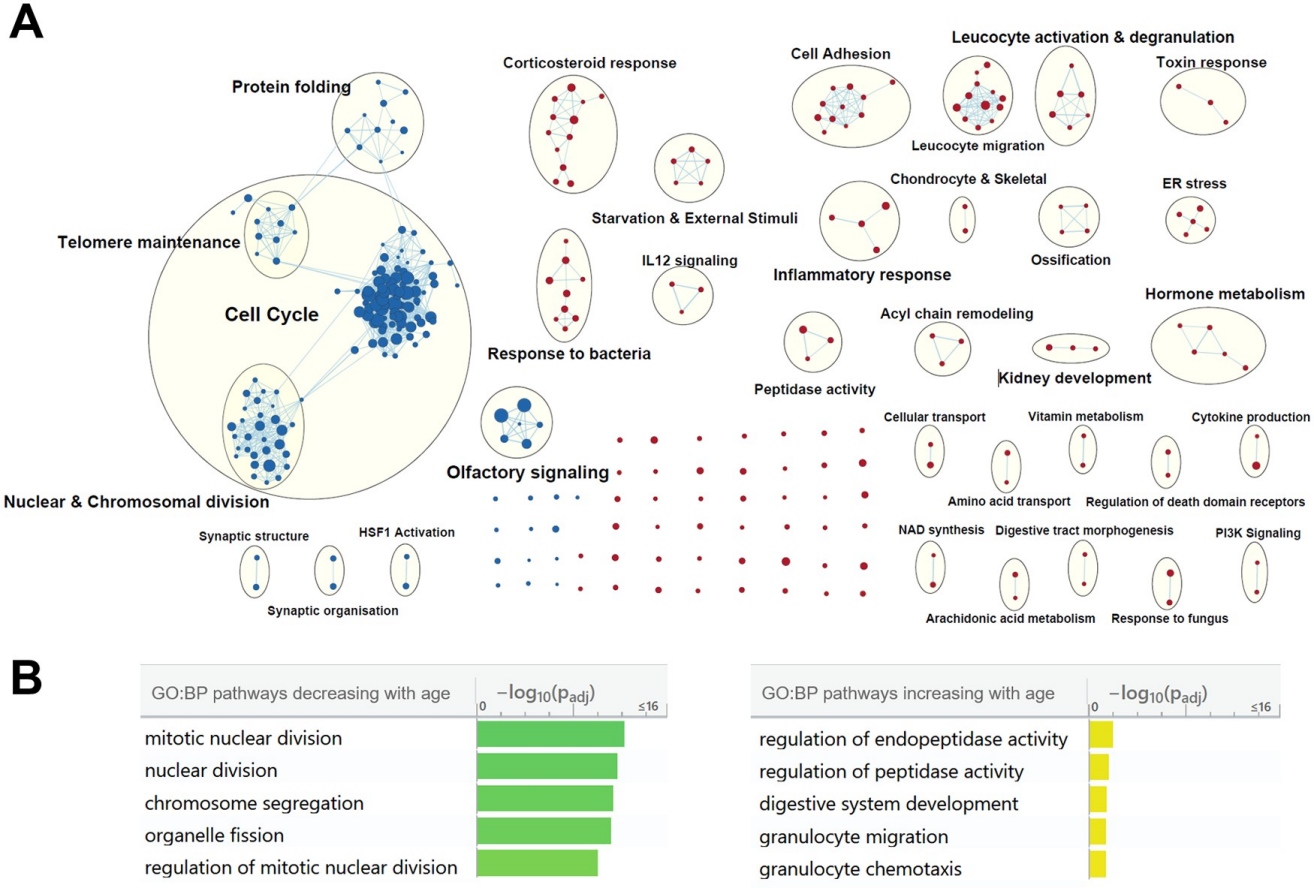
Pathways associated with aging. **A** EnrichmentMap clusters of the gene set enrichment analysis using Gene ontology: biological processes and REACTOME databases. Red nodes are upregulated gene sets with increasing age. Blue nodes are downregulated. Node size is increased based on normalised enrichment score (NES) with the node size increasing from NES 1.5 to NES 2.5. **B** Top 5 enriched gene sets from pathway enrichment analysis using g:profiler and gene sets from GO: biological processes.

Gene set enrichment analysis (GSEA) identified 317 gene sets that correlated significantly with age (FDR <10%). 157 were upregulated and 160 downregulated with increasing age, respectively (S3 Table). Gene sets were clustered using EnrichmentMap (Fig 3A). Downregulated gene sets primarily concerned different aspects of mitosis and the cell cycle. However, several highly enriched gene sets also covered olfactory receptors and G-protein coupled receptor signaling. Upregulated gene sets were much more diversely distributed across processes with clusters such as ER stress, response to corticosteroids and developmental pathways e.g. for kidney and bone. Three clusters were annotated for leucocyte activation and migration; however, transcripts for general markers of immune cells were not detected (CD11, CD19, CD45) or were not significantly altered by age (CD3, CD4, CD8, CD57, CD15b and FCER1A).

Genes significantly altered by age (FDR >20% and with log2 fold change >1.5 across 80 years of age) were used for functional enrichment analysis using g:Profiler. Downregulated genes were again enriched for gene sets concerning mitosis, in total 83 gene sets were significantly downregulated with age (g:SCS adjusted p-value <0.05). 6 gene sets were significantly upregulated with increasing age(g:SCS adjusted p-value <0.05). Two pathways concerned peptidase activity, however, transcript levels of PC2 and PC1/3, two important peptidases for endocrine function did not correlate with age. The five most significant gene sets of each phenotype are presented in Fig 3B.

### Validation of earlier findings on the aging transcriptome of islets of Langerhans

**Fig 4:**
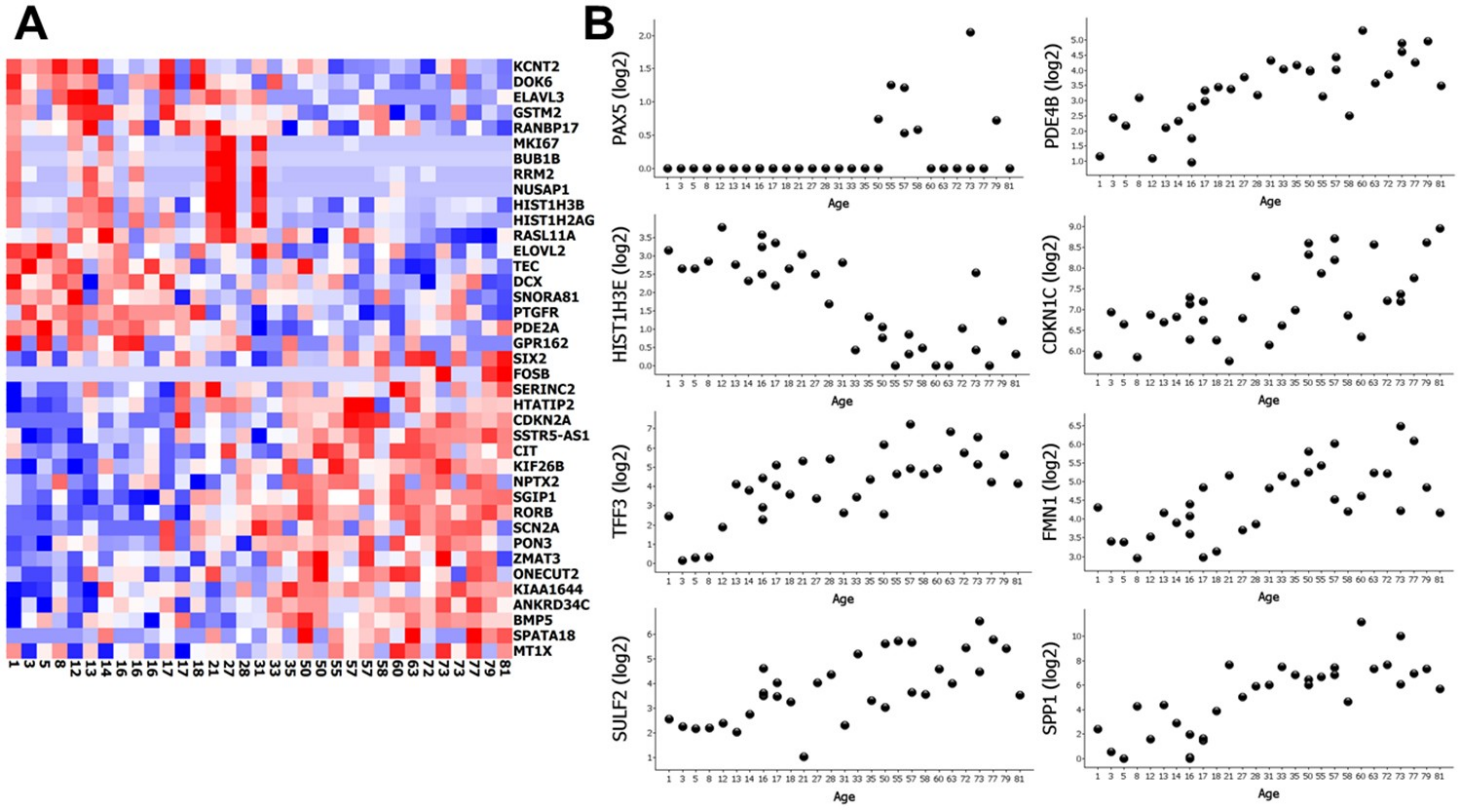
Comparison of differentially expressed genes from a previous study. **A** Heat map of the 39 differentially expressed genes overlapping between the study by Arda et al. (comparing isolated islets from old and young donors) and our data (genes that correlated with age in our generalized linear model (FDR<20%) in islets extracted by LCM from donors of increasing age. **B** Scatter plots of the 8 most significantly differentially expressed genes in our model that did not overlap with the differently expressed genes in Arda et al. Gene counts are presented as log2 values of normalized gene counts. In (A), each column represents one donor and age is noted below.

Arda et al. [18] compared the transcriptome of islets isolated by enzymatic digestion of the pancreas from children to that of adult islets and found 567 genes that co-varied with age. 344 of those genes were present in our material after filtering (i.e. were detected in at least 5 samples). 39 genes correlated with age in our generalised linear model (FDR<20%) (Fig 4A). However, three genes (*FOSB*, *CIT* and *MT1X*) were differentially expressed in the opposite direction with increasing age when compared to the results by Arda et al. Among the most differentially expressed genes that overlapped with findings of the Arda study were *FOSB*, *RORB* and *SSTR5-AS1*, but also 8 genes novel to this study; *PAX5*, *HIST1H3E*, *TFF3*, *SULF2*, *PDE4B*, *CDKN1C*, *FMN1* and *SPP1*. The novel genes are presented as scatter plots in Fig 4B.

### Genes linked to type 2 diabetes that also correlate with age

**Fig 5:**
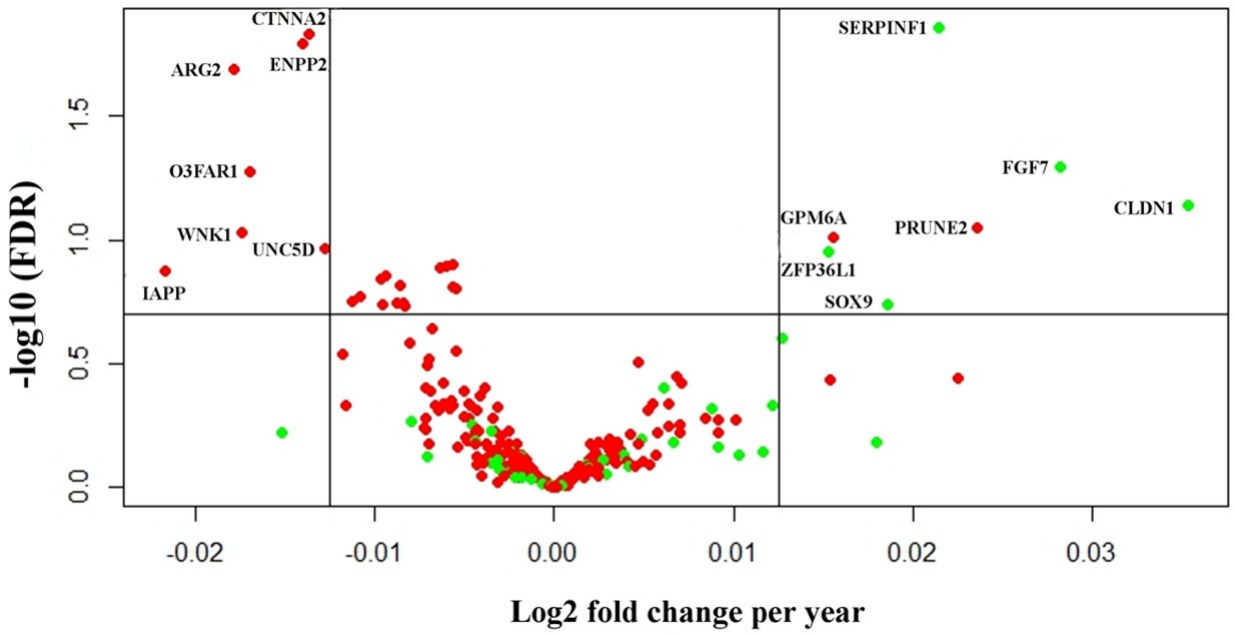
Genes associated with T2D that co-vary with age. Volcano plot of genes reported to be upregulated (green) or downregulated (red) in islets from subjects with type 2 diabetes in at least two studies. On the X-axis is log2 fold change per year, and the threshold is set at 0.0125, corresponding to a log2 fold change of 1 across 80 years of life. On the Y-axis there is a threshold corresponding to FDR 20%.

Several studies have compared the transcriptome between non-diabetic and type 2 diabetic subjects either in islets [30–33] or in dissociated single islets cells [34, 35]. In the literature, we found 276 genes reported in at least two published data sets to be differentially expressed in type 2 diabetes. 252 of which passed filtering in our data set. Using an FDR threshold of ≤20% and log2 fold change of 1 across 80 years of life, 7 of the genes reported to be downregulated in T2D decreased with age in our study; *CTNNA2*, *ENPP2*, *ARG2*, *O3FAR1*, *WNK1*, *IAPP* and *UNC5D*, while two of the genes increased with age; *GPM6A* and *PRUNE*. 5 of the genes reported to be upregulated in T2D increased with age, *SERPINF1*, *FGF7*, *CLDN1*, *ZFP36L1* and *SOX9* (Fig 5B and S4 Table).

## Discussion

Using pancreatic islets from deceased organ donors of ages that covered the human lifespan we found 346 genes that co-varied with increasing age. Enrichment analysis saw pathways relating to mitosis decrease with age. As in previous studies, we also found *CDKN2A*, a marker of senescence, to be upregulated with increasing age, together with other cell-cycle inhibitors *CDKN1A* and *CDKN1C*. Senescence in beta cells has been linked to improved insulin secretion in mice through p16INK4A leading to increased glucose metabolism due to higher levels of *AldoB* and higher numbers of mitochondria through *Ppargc1a*, a transcription factor controlling mitochondrial biogenesis [36]. Both these genes were significantly upregulated in our material suggesting that this mechanism might be similar in humans.

Our findings corroborate the prevailing view that beta cells attain a senescent phenotype with age with a corresponding downregulation of the cell cycle machinery. However, as cellular division of beta cells postnatally is rare at all ages, we cannot exclude that a significant downregulation of the cell cycle machinery could be contributed from other parts of the islet such as endothelial cells. The number of Ki67-expressing beta cells has been seen to increase in organ donors with prolonged stay at intensive care units prior to organ procurement [37]. These cells might represent a subpopulation of cells still capable of proliferation [38]. Even if only a fraction of a percent of the islet cells divide at a given time this would renew the entire endocrine pancreas in a matter of years [39].

The comparison between data obtained in the current study and that by Arda et al. [18] showed some divergences. The study by Arda et al. had a study design able to detect the changes in the transcriptome between children and adults, while the current study was designed to detect changes across decades of life. Limitations in the current study is that we have very limited access to donor medical history due to patient confidentiality and that scarcity of organ donor pancreata in the lower age groups did not allow full matching on gender. Laser capture microdissection (LCM), as used in this study, leads to relative low levels of RNA collected as well as the interrogation of the entire islets instead of specific endocrine cell types. Therefore, these findings cannot with certainty be attributed to any one cell type. On the other hand, using LCM prevents any changes in the transcriptional profile during islet isolation, culture and dissociation into single cells, instead interrogating the tissue as close to the physiological state as possible. Indeed, transcriptional analysis of LCM material cluster together when compared to isolated islets, regardless of if the initial tissue comes from organ donors or surgical resection, suggesting that methodology for how the tissue is treated is more important than how the tissue is acquired [31].

One of the most differently expressed among our novel genes found to be upregulated with age was *SPP1*, coding for osteopontin, a protein that has been linked to both protection from IL1-beta mediated cytotoxicity [40] as well as hyperglycemia [41]. While this upregulation cannot be specifically linked to beta cells in this study, it is interesting to speculate that an age-correlated upregulation of *SPP1* could have a protective effect, contributing to why type 1 diabetes pathogenesis progress slower with higher age at onset [42].

Big gene sets of olfactory receptors were found to be downregulated with age using GSEA. Olfactory receptors are G-protein coupled receptors and have previously been found in beta cells. They can potentiate glucose-stimulated insulin secretion based on recognition of different nutrients [43]. Lower levels of olfactory receptors could be detrimental to beta cell function.

Senescence-associated secretory phenotype (SASP) has been suggested as a cause of loss of function of beta cells in both type 1 and type 2 diabetes [44, 45]. SASP beta cells are characterized by IL-6 production together with expression of *CDKN1A*, *SERPINE1*, *MMP2*, *CCL4*, *IL1A*, *CXCL10*. *CDKN1A* and *SERPINE1* were upregulated by age in our study. Of the remaining genes, only *MMP2* was expressed in our data set and was not correlated to age, which run counter to an increase of SASP-cells with age. However, our donors were all selected to be normoglycemic, which could bias us towards healthier islets.

Transcriptional studies on type 2 diabetic islets or islet cells showed surprisingly little overlap, most likely due to differences in methodology. Among the 252 genes validated by at least one other study, a few (12/252) were differentially expressed in the same direction with increasing age. This could be interpreted as that general age-related changes to the islet transcriptome is not an underlying driving force of the increased prevalence of type 2 diabetes with increasing age.

To the best of our knowledge, this study represents the first time that the aging transcriptome of human islets of Langerhans has been interrogated without prior digestion and culture and complements previous studies on bulk islets and single cells. In line with previous findings in mice and humans, we see an increased transcription of genes linked to senescence and a downregulation of the cell cycle machinery on the whole islet level. We also present novel genes linked to the aging islet, such as *SPP1*, which potentially could affect how the beta cell reaction to stress changes with age.

## Supporting information

S1 Table Normalized counts of filtered genes. 14,794 genes from 33 donors.
(XLSX)

S2 Table DEG analysis of genes change with age. Log fold change corresponds to change over 1 year.
(XLSX)

S3 Table Results of gene set enrichment analysis (GSEA). List of all gene sets analyzed.
(XLSX)

S4 Table DEG analysis of genes associated with T2D. (XLSX)
